# Three species in one: a revision of *Clemensiaalbata* Packard (Erebidae, Arctiinae, Lithosiini)

**DOI:** 10.3897/zookeys.788.26048

**Published:** 2018-10-08

**Authors:** B. Christian Schmidt, J. Bolling Sullivan

**Affiliations:** 1 Canadian National Collection of Insects, Arachnids and Nematodes, Agriculture and Agri-Food Canada, K.W. Neatby Bldg., 960 Carling Ave., Ottawa, ON, Canada K1A 0C6200 Canadian National Collection of Insects, Arachnids, and Nematodes Ottawa Canada; 2 Craven St., Beaufort, North Carolina 28516, USA Unaffiliated Beaufort United States of America

**Keywords:** algivory, cryptic species, lichen moth, lichenivory

## Abstract

*Clemensiaalbata* Packard, previously thought to be a single, widely distributed North American species, is here shown to consist of three distinct, primarily parapatric species: *Clemensiaalbata* Packard, *C.umbrata* Packard, **stat. rev.**, and *Clemensiaochreata* Schmidt & Sullivan, **sp. n.** Adults and genitalic structures of the three species are illustrated.

## Introduction

The genus *Clemensia* Packard encompasses about 55 described species (Seitz 1919–40; Gibeaux 1983; Gibeaux 1988), in addition to numerous undescribed Neotropical species (Sullivan and D. Janzen unpubl. data). There is no modern review of the genus, and the systematic placement of the genus tentatively remains in the tribe Cisthenini (Bendib and Minet 1999). *Clemensiaalbata* Packard has generally been the only species attributed to the fauna of the United States and Canada (Franclemont 1983), although Ferguson and Opler (2006) recently recognized a second species, *C.patella*. This species was, however, wrongly attributed to the Pacific Coast fauna (Schmidt and Opler 2008) stemming from a typographical error in Ferguson’s manuscript after his death (Lafontaine and Schmidt 2010). This southeastern U.S. species has subsequently been referred to *C.patella* “of authors” (Lafontaine and Schmidt 2010).

The variability of *Clemensiaalbata* was recognized as early as Seitz (1919), who stated that it is “sometimes of a pure, sometimes dull whitish-grey, clouded or spectacled, has, according to its coloring, received five different names.” Packard (1864, 1872) described eastern and western North American *Clemensia* as separate species, and although the two names were long ago synonymized, Packard’s initial assessment nearly 150 years ago would prove correct. More than 40 years of collecting *Clemensia* in North Carolina by the second author revealed the presence of three phenotypes, and subsequent examination and comparison of eastern North American specimens, together with analysis of DNA barcode sequences (Zahiri et al. 2017), indicate that three separate species occur in North America. The purpose of this paper is to diagnose and illustrate these three species in an effort to clarify what is currently recognized as one variable species, *Clemensiaalbata* Packard.

## Materials and methods

*Repositoryabbreviations*. Voucher specimens (Suppl. material 1) are deposited in the following collections:

**BIOUG** Centre for Biodiversity Genomics, University of Guelph, Guelph, Ontario

**NHML** Natural History Museum, London

**CNC** Canadian National Collection of Insects, Arachnids, and Nematodes, Ottawa, Ontario

**JBS** J. Bolling Sullivan Collection, Beaufort, North Carolina, USA

**JTT** Jim T. Troubridge Collection, Hagersville, ON

**MEM** Mississippi Entomological Museum, Starkville, MS

**NOFC** Northern Forestry Centre, Canadian Forest Service, Edmonton, AB

**PFC** Pacific Forestry Centre, Canadian Forest Service, Victoria, BC

**RBCM** Royal British Columbia Museum, Victoria, BC

**USNM** National Museum of Natural History, Washington, District of Columbia, USA

Due to the difficulty in species identification based solely on photographs, records from citizen-science groups such as BugGuide.net and iNaturalist.org were not included here, with the exception of a few records that could be identified with reasonable confidence, and that represent significant range-gap or range-edge records (Suppl. material 1). Procedures for dissecting and preparing genitalia follow those of Lafontaine (2004). Genitalia were photographed using a Leica M205C microscope and DFC450 camera, and processed using Leica Application Suite 4.8 and Adobe Photoshop. DNA extraction, PCR amplification, and sequencing of the COI barcode region were performed at the Canadian Centre for DNA Barcoding and followed standard protocols (Hebert et al. 2013; http://www.ccdb.ca/resources.php). Resulting data were managed and analyzed using BOLD (Barcode of Life Data Systems; Ratnasingham and Hebert 2007), available at http://v4.boldsystems.org/. Mitogenomic divergence was calculated based on Kimura 2-Parameter (K2P) distances of COI barcodes. Variation of the ‘barcode’ section of the COI gene was compared among 150 specimens from across North America (Suppl. material 1). Only sequence records greater than 500 bp (range 500 bp–658 bp) are included. Sequence comparisons were generated on the BOLD website with the following parameters: Distance Summary Model: Kimura 2 Parameter; Deletion Method: Pairwise Deletion; Alignment: BOLD Aligner (Amino Acid based HMM).

## Results

**Figures 1–12.**
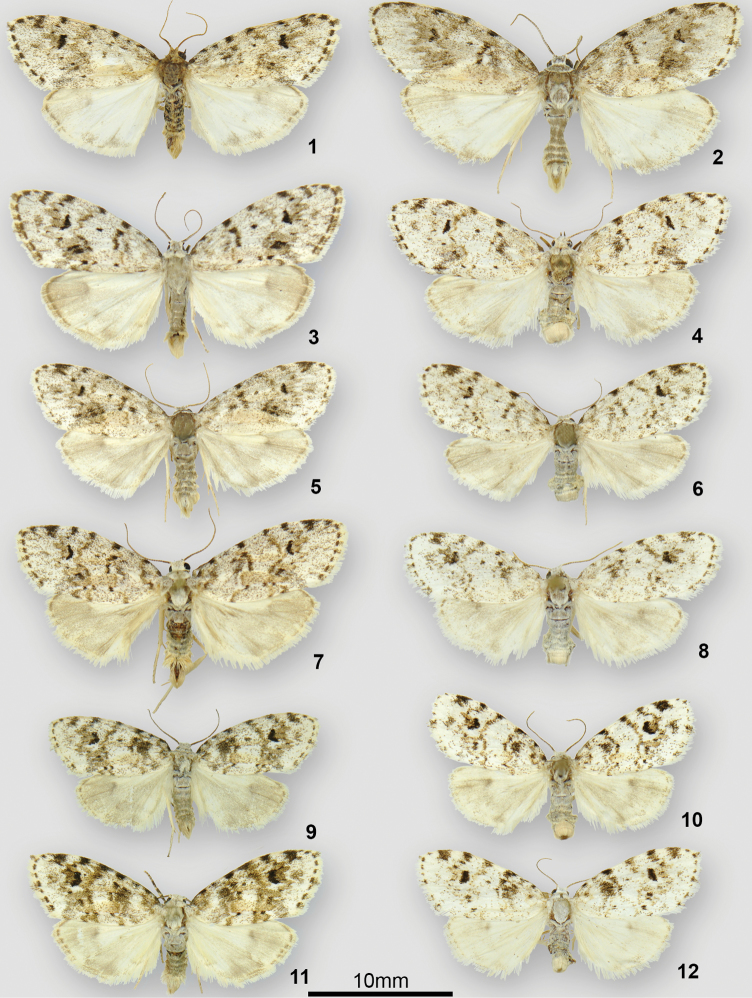
*Clemensia* adults. **1–4***C.albata*, **1** ♂, Manitoulin Island, Ontario, Canada **2** ♂, Grandfather Mountain, 4850’, Avery Co., North Carolina, USA **3** ♂, Langley, British Columbia, Canada **4** ♀, Tweed, Ontario, Canada **5–8***C.umbrata*, **5** ♂, Backus Woods, Ontario, Canada **6** ♀, Backus Woods, Ontario, Canada **7** ♂, Starkville, Oktibbeha Co., Mississippi, USA **8** ♀, Barksdale A.F.B., Bossier Parish, LA, USA; **9–12***C.ochreata*, **9** ♂, Gainesville, Paynes Prarie State Park, Alachua Co., Florida, USA **10** ♀ holotype, Anthony, Marion Co., Florida, USA **11** ♂, Middleton Creek, Franklin Co., Mississippi, USA **12** ♀, Sweetbay Bogs, Stone Co., Mississippi, USA.

The three *Clemensia* phenotypes that occur in North America are shown in Figs 1–12. An initial comparison based on specimens from North Carolina, where the three phenotypes co-occur, permitted a calibrated comparison across eastern North America. This revealed that “*patella* of authors” (in the sense of Lafontaine and Schmidt 2010) is found in the coastal plain from North Carolina south into Florida and west to Texas. This taxon is described herein as *C.ochreata* sp. n., the small, pale phenotype here determined to represent true *C.albata* is found throughout North Carolina from the coast up to 1400 m in the mountains. It is the most common and widespread *Clemensia* in eastern North America, occurring from southern Quebéc and Ontario to at least Georgia, Oklahoma and Missouri. The third phenotype is restricted to higher elevations in the southern Appalachians, and occurs from 940 m and above throughout the mountains of North Carolina. It replaces *C.albata* to the north, and is found across the boreal forest region from Nova Scotia to the Pacific Northwest. This boreo-Appalachian taxon is *C.umbrata*. *Clemensiaumbrata* and *C.albata* overlap in distribution throughout much of the Northeast, but are usually distinguishable based on phenotype, size, and flight period as detailed below in the Diagnosis sections.

Examples of all three phenotypes resolved into three distinct DNA barcode sequence clusters, or BINs (Barcode Index Numbers; see Ratnasingham and Hebert 2013), each containing samples from differing but partially overlapping regions of North America (Figs 19–21, 23). Divergence between *C.umbrata* and *C.albata* ranges from 1.71–2.89 %, with maximum divergence within *C.umbrata* at 0.90 %. Divergence between *C.ochreata* and *C.albata* ranges from 2.18–3.79 %, compared to a maximum of 0.92 % within *C.ochreata* (Figure 23). Barcode sequence clusters for broadly overlapping *C.umbrata* and *C.albata* in Ontario and Quebéc corroborated the phenology differences between the two taxa, with univoltine *C.umbrata* primarily in July, and bivoltine *C.albata* mostly in June and late August.

### Clemensia umbrata

Taxon classificationAnimaliaLepidopteraErebidae

Packard stat. rev.

Figs 1–4 , 13 , 16

Clemensia umbrata Packard, 1872: 85. Type locality. Congress Springs, Santa Clara Co, California [lost] male holotype. Note. The type locality was given as “California” in the original description, and Edwards (1874) later writes that the only type was destroyed in the mail when Packard returned it, and clarifies the source of the type material as “Congress Springs, Santa Clara County.”
Clemensia irrorata H. Edwards, 1874, p.185. Type locality. “Victoria, V.I. [Vancouver Island, British Columbia]”

#### Diagnosis.

*Clemensiaumbrata* is most similar to *C.albata*; flight time and locality aid in separating the two. Both species occur together only from southern Quebéc and eastern Ontario southward; *C.umbrata* is the only *Clemensia* species across the boreal forest region and the Pacific Northwest (Figure 19). Where the range overlaps that of *C.albata*, the phenology differs in that *C.umbrata* is univoltine with adults in July and early August (as early as June in the southern Appalachians) (Figure 22), whereas *C.albata* is bivoltine in the Northeast and possibly multivoltine farther south. In northeastern North America the flight peaks of *C.albata* are in mid-June and late August largely outside that of *C.umbrata* (Figure 22), but the flight periods of the two overlap in late July and possibly early August. In the eastern US *C.umbrata* becomes increasingly restricted to higher elevations southward, whereas *C.albata* is more widespread. For example, in North Carolina *C.umbrata* is usually found above 3100’ whereas *C.albata* occurs below 4600’. Similar habitat/ecozone segregation likely occurs elsewhere, but further study is needed.

Externally, *C.umbrata* differs from *C.albata* in its larger size in regions of sympatry (northern boreal *C.umbrata* are smaller and not noticeably significantly larger than *C.albata*), with male forewing length of 12.3 mm (n = 9) versus 10.8 mm (n = 6) in *C.albata*. Wing pattern differences are difficult to discern, especially flight-worn individuals, but *C.umbrata* has a more contrasting forewing pattern that is more suffused with grey and black, and often with a diffuse dark grey postmedial patch near the anal margin; this patch is absent or much more restricted in *C.albata*.

**Figures 13–15.**
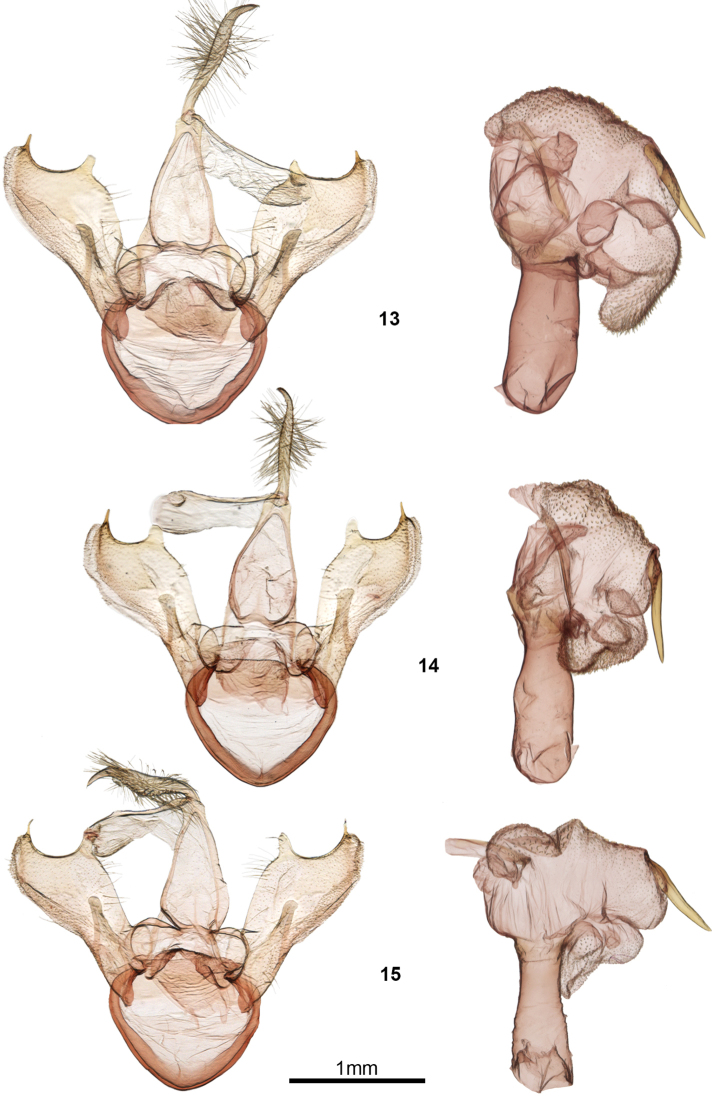
*Clemensia* male genitalia. **13***C.umbrata***13a** ON, Carp Ridge, CNC gen. prep. #16762 **13b** ON, Manitoulin Is., CNC gen. prep. #16763 **14***C.albata* ON, Backus Woods, CNC gen. prep. #16759 **15***C.ochreata* S.C., The Wedge Plantation, CNC gen. prep. #14769.

**Figures 16–18.**
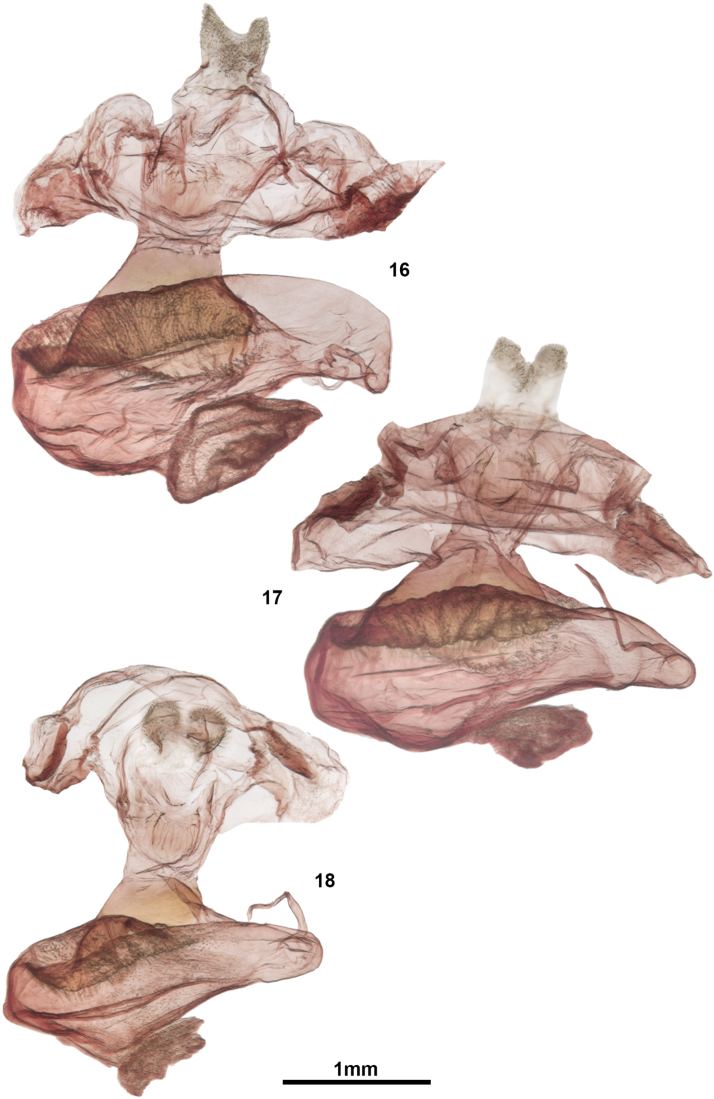
*Clemensia* female genitalia. **16***C.umbrata* MB, Spruce Woods, CNC gen. prep. #14772 **17***C.albata* ON, Backus Woods, CNC gen. prep. #14773 **18***C.ochreata* FLA, Dunedin, CNC gen. prep. #14774.

Internally, the male genitalic structure of *C.umbrata* and *C.albata* differs in the shape of the basal ventral diverticulum of the vesica, which is bilobed in *C.umbrata* versus heart shaped in *C.albata* (Figs 13, 14). The female corpus bursae of *C.umbrata* (Figure 16) is less elongate with longer internal spinules and a more broadly joined appendix bursae compared to *C.albata* and *C.ochreata*.

#### Biology.

Dyar (1904) describes the egg and first two instars based on samples from southeastern British Columbia, stating that larvae overwinter (as second instar?). The egg is unusually large with a diameter of 0.8 mm. The eggs are covered with setae from the female abdominal tip. McCabe (1981) described the larval biology, but it is unclear if his account is referable to *C.albata* or *C.umbrata*. Larvae probably graze algae growing on tree bark and possibly other substrates according to McCabe (1981), but both Dyar (1904) and Miller and Hammond (2000) state that larvae feed on lichens; a larva likely referable to *C.umbrata* was found on white birch cut for firewood in Renfrew Co, Ontario in late June (J. Dombroskie, pers. comm.). Miller and Hammond (2000) report this species as feeding in lichens on trees and large shrubs in the Pacific Northwest, especially on gary oak. *Clemensiaumbrata* was collected 74 times during the Canadian Forest Insect Survey, always from conifers, and mostly from white spruce (49/74 collections; McGugan 1958). This may however indicate larvae feeding on algae-encrusted conifer twigs, whereas algal growth is usually limited to thicker branches and trunks of birch, where larvae are less likely to be collected by conventional sampling methods. *Clemensiaumbrata* is univoltine throughout its range, with peak adult abundance in late July in northeastern North America (Figure 22), but appearing as early as June in the southern Appalachians. In the boreal region the larva is present from mid-May to mid-July (presumably having overwintered as a second or third instar), and with most collections from mid-June (McGugan 1958).

#### Distribution.

**Figures 19–21.**
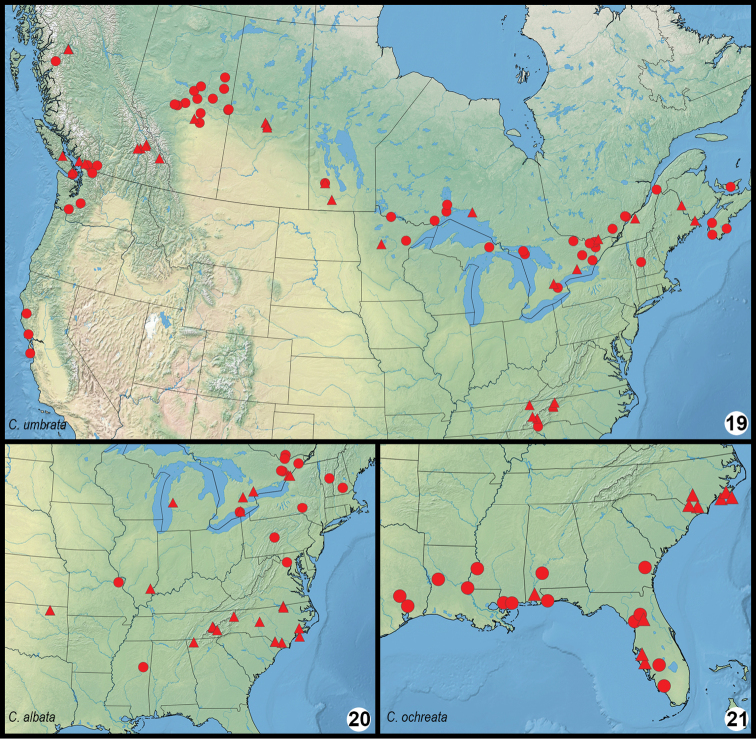
Distribution of examined material for *Clemensia* species (circles), including DNA barcode vouchers (triangles). **19***C.umbrata***20***C.albata***21***C.ochreata*.

*Clemensiaumbrata* occurs from Nova Scotia across the boreal region to the Pacific coast, southward into central California and northern Idaho (Figure 19; Pacific Northwest Moths website). The northernmost records are for north-coastal British Columbia (Figure 19), southernmost Northwest Territories (southwest of Hay River; McGugan 1958), and Havre-St.-Pierre, Québec (Handfield 2011). *Clemensiaumbrata* is absent from the entire central and southern Rocky Mountain region of the USA. The extent of distribution in the eastern US is still poorly defined; minimally, *C.umbrata* occurs in the northern Great Lakes region, Vermont, and the southern Appalachians (Tennessee and North Carolina), but it is likely more widespread in the Appalachians.

#### Remarks.

As defined here, *C.umbrata* represents the taxon that occurs across most of Canada and western USA that was previously called *C.albata*. In most of its range (except eastern North America), it is the only *Clemensia* species. Although no California specimens of *C.umbrata* were available for DNA analysis, examined California material was not distinguishable morphologically from that of the Pacific Northwest, with the latter genetically very similar to the transboreal/Appalachian taxon. The distribution of *C.umbrata* is continuous along the Pacific coast from southern British Columbia to central California (Figure 19; see also Pacific Northwest Moths website), and we accordingly treat all as a single species under the name *umbrata*.

### Clemensia albata

Taxon classificationAnimaliaLepidopteraErebidae

Packard

Figs 5–8 , 14 , 17

Clemensia albata Packard, 1864, p. 101. Type locality. “Norway, Me. (Mus. Comp. Zool., Smith), Brunswick, Me., August.” [unknown]. Note. The holotype of albata should be with other Packard types at MCZ, but the online inventory of MCZ types (mczbase.mcz.harvard.edu) indicates it is not, and as such iot may no longer be extant.
Uxia albida Walker, 1866, p. 1897. Type locality. “North America;” female type [NHML]. Note. The holotype is a female, mistakenly believed to be a male by Walker, and therefore described as a new species in a separate genus as Repacana. The holotype is a small, poorly-marked specimen typical of female C.albata. The type locality is unspecified but is likely the northeastern US.
Repa cana Walker, 1866, p. 1898. Type locality. “United States;” male type [BMNH]. Note. The holotype is a male without an abdomen.

#### Diagnosis.

*Clemensiaalbata* is the most common *Clemensia* in most of eastern North America south of the boreal forest region. In the northeastern US and the Appalachians, *C.albata* can be confused with *C.umbrata*, and a differential diagnosis is presented in the *C.umbrata* account. Along the Atlantic coastal plain from North Carolina to Florida and across the southern states to at least Mississippi, *C.albata* can occur with *C.ochreata*, and the two can be difficult to separate. *Clemensiaalbata* usually differs from *C.ochreata* in its pure white rather than ochre-white ground colour, less prponounced dark spots along the forewing costa, a less distinct antemedial dark patch, lack of a well-defined ventral hindwing medial band, and narrower uncus (Figure 14). *Clemensiaalbata* is slightly larger than *C.ochreata*, but there is overlap, with an average forewing length of 10.77 mm for *C.albata* (n = 6) versus 9.83 mm for *C.ochreata* (n = 9) (first brood, barcoded specimens only). Habitat, phenology, and larvae may also differ, but further research on the comparative biology of *C.albata* and *C.ochreata* is needed. Internally, the male genitalic structure of *C.albata* differs from that of *C.ochreata* in the shape of the basal ventral diverticulum of the vesica, which is heart shaped in *C.albata* versus bilobed in *C.ochreata*. The field of spicules on the basal lobe is smaller, and the cornutus relatively shorter in *C.albata*.

#### Biology.

**Figure 22.**
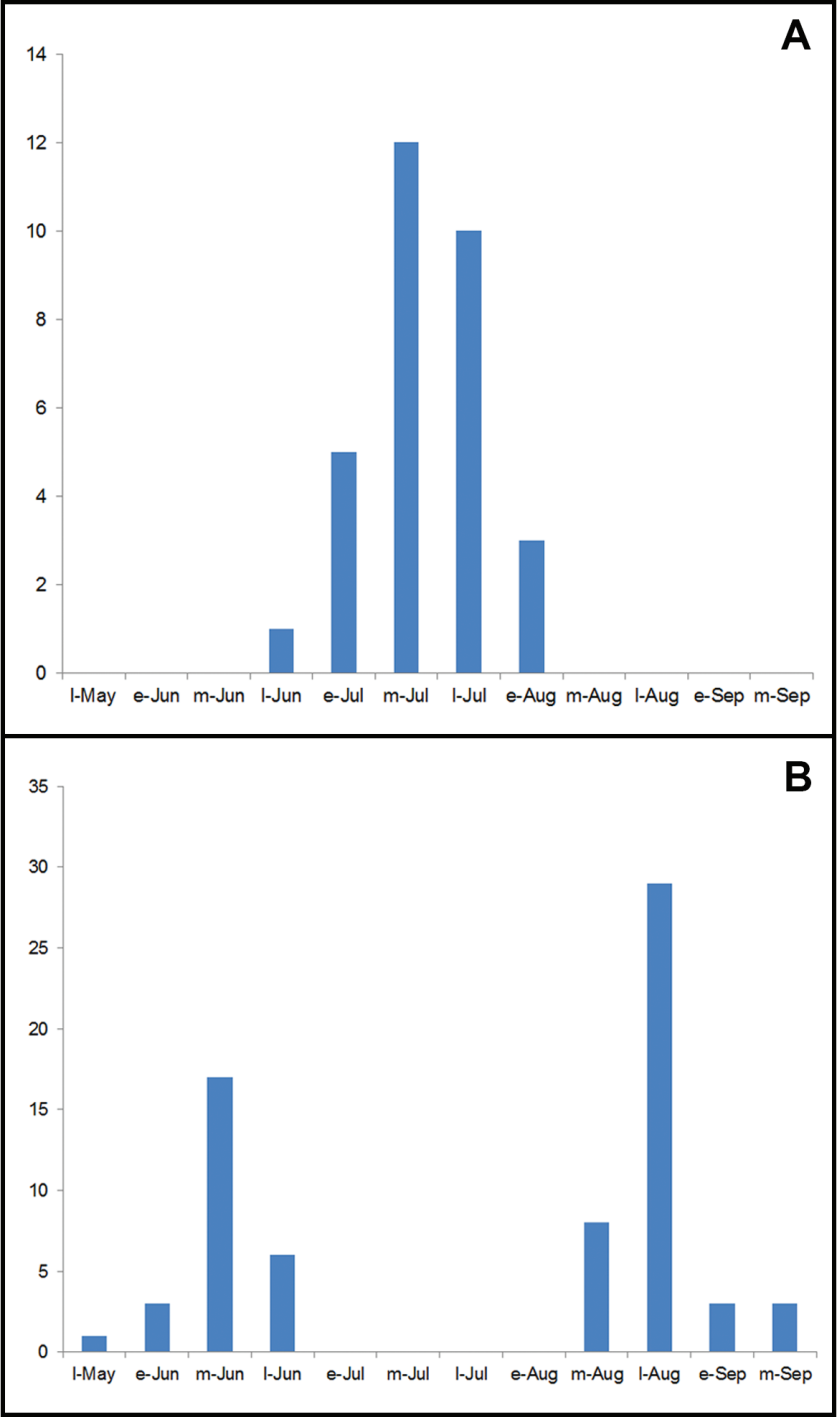
Comparative adult flight times of *Clemensiaumbrata* and *C.albata* in northeastern North America, based on collection dates by 10-day monthly intervals.

The larvae feed on blue-green algae and lichens growing on tree trunks (Wagner 2005) along with a number of other Lithosiini and small noctuids (Wagner et al. 2011). Color morphs or pattern variability of larvae has not been recorded. The life history data and larva illustrations presented by Wagner (2005) and McCabe (1981) probably apply to this species, but these need to be re-evaluated in light of the current taxonomic results. *Clemensiaalbata* is bivoltine in the northeast with peak abundance in mid-June and late August (Figure 22), and probably multivoltine in the southern US. In North Carolina it is widely distributed from the coast up to 4600’ in the mountains, and occurs from March until September.

#### Distribution.

Examined specimens identified with certainty as *C.albata* are mapped in Figure 20, and this species occurs at least from eastern Ontario and southernmost Quebéc through New England southward to Georgia, Mississippi and Oklahoma. The northwestern range limit is uncertain but is likely in the western Great Lakes region. Specimen photographs from south-central Minnesota (Wright Co.) in mid-June (butterfliesandmoths.org, record # 978800) and central Wisconsin in early August (butterfliesandmoths.org, record # 1097249) are consistent with *C.albata*, but voucher specimens should be examined.

#### Remarks.

There is some uncertainty in the identity of the holotype of *Clemensiaalbata*, given the type locality and the similarity to *C.umbrata*, but two facts help in ascertaining what taxon the name *albata* applies to: the types were collected in August in southern Maine, pointing to second-brood specimens of the bivoltine eastern species (versus univoltine *C.umbrata* flying mostly in July), and the wing expanse given as 0.83 inch, or 21.1 mm, which is smaller than most eastern *C.umbrata*. Examined specimens from regions adjacent to the *C.albata* type locality in southern Maine (Figure 20) have also so far proven to be the smaller, bivoltine species.

### Clemensia ochreata sp. n.

Taxon classificationAnimaliaLepidopteraErebidae

http://zoobank.org/28C3DAC5-7EDB-4B45-AEDD-28AC3572D68D

Figs 9–12 , 15 , 18

#### Type material.

**Holotype** ♀ (Figure 1). Florida: Marion Co., W. Anthony Rd., 1.4 mi. WSW Anthony, 29°17'N 82°08'W, 13.Jan.2007, T.S. Dickel, DNA voucher # CNCNoctuoidea13909 [CNC]. **Paratypes** 49♂ 28♀ **Alabama**: Monroe Co., Haines Island Park, 4.Apr.1995, J. A. MacGown, 1♂; **Florida**: same data as holotype, 4.Jan.2007, 1♀, 15.Mar.2006, 1♂; [Pinellas Co.,], Dunedin, 22.Mar.1999, J.G. Filiatrault, CNC genitalia slide # 14774, 1♀; [Alachua Co.], Gainesville, 13.Mar.2013, C. Belanger, 2♂; Levy Co., Goethe State Forest, Cow Creek Rd., 10.Feb.2012, T.S. Dickel, 1♀; DeSoto Co., Nocatee, 27°10.07'N 81°54.63'W, 23.Feb.2014, J. Troubridge, 1♂; same locality and collector as previous, 11.Mar.2011, 9♂ 1♀; 20.Mar.2012, 1♂; 1.Apr.2012, 1♂; 3Apr.2011, 2♂; 3.Apr.2013, 6♂; 2.Apr.2010, 2♂ 1♀; 23.Apr.2011, 1♂; 14.Apr.2010, 1♀; 23.Feb.2012, 2♂; 30.Nov.2010, 1♂; 18.Jan.2012, 2♂. Monroe Co., Dagny Johnson State Park, 25.165°N 80.362°W, 22.Mar.2012, J. Troubridge, 1♀. Dixie Co., Hwy 361, 29.564°N 83.380°W, 5.Apr.2016, J. Troubridge, 1♀. Okeechobee Co., Kissimmee Prairie State Park, 27.584°N 81.044°W, 27.Mar.2013, 1♀, 5.Feb.2014, 3♂. Collier Co., Fakahatchee Strand State Park, 25.98°N 81.41°W, 4.Feb.2014, J. Troubridge, 3♀; 21.Dec.2011, 1♀; 23.Mar.2015, 1♂; 21.Feb. 2014, 2♀; 15.Jan.2012, 1♂. Sarasota Co., North Port, 27°02.5'N 82°05.0'W, 29.Nov.2012, J. Troubridge, 1♀; 2.Feb.2011, 1♂; 27.Nov.2011, 1♂; 8.Jan.2012, 1♂; 28.Mar.2012, 1♀; 24.Nov. 2014, 1♂. **Georgia**: Long Co., 3 mi. SW Ludowici, Griffin Ridge, 6.Mar.2008, C. Schmidt & J. Adams, 1♀. **Mississippi**: Stone Co., Sweetbay Bogs, T2S R13W Sec 34SW, 12.Mar.1991, D.M. Pollock, 1♀; Franklin Co., Middleton Creek, T5N R3E Sec.21E, 7.Apr.1992, J. MacGown, T. Schiefer, 1♂; Hancock Co., Stennis Space Center, 21.Mar.1994, R. Kergosien, 1♂; Harrison Co., Long Beach, 20.Mar.1995, R. Kergosien, 1♂; same data as previous, 17.Mar.1996, 1♂; Wilkinson Co., Clark Creek Nat. Area, 10.Mar.1989, T. Schiefer & J. MacGown, 1♂; Claiborne Co., 3.6 mi W Port Gibson, 12.Jul.1993, D. M. Pollock, 1♀; **South Carolina**: [Charleston Co.], The Wedge Plantation, McClelanville, 7.Jun.1977, E.G. & I. Munroe, CNC genitalia slide #14768, 1♂. **North Carolina**: Jones Co. Croatan Natl. Forest, Haywood Landing, 4.May.2008, J. Bolling Sullivan, DNA voucher # 09-NCCC-155, 1♂; Columbus Co., Lake Waccamaw St. Pk., April 16, 2010, J. Bolling Sullivan, DNA voucher # 10-NCCC-281, 1♂. [CNC, MEM, JBS, JTT]

#### Etymology.

The name *ochreata* is a noun in opposition and refers to this species’ characteristic ochre forewing tint.

#### Diagnosis.

Very similar to *Clemensiaalbata*, but differing from that species by the smaller mean forewing length, more extensive, and brighter ochreous scales along the forewing antemedial and postmedial lines; overall more contrasting pattern, especially the heavier costal dark spots (most pronounced on the forewing underside), and the more prominent and better-defined medial dark patch basad of the antemedial line. Internally, the basal diverticulum of the male vesica has much smaller spicules than in *C.albata*. The valve shape is proportionally shorter and stouter than that of *C.albata*. In females, the corpus bursae is more elongate than that of *C.albata* and with shorter spinules lining the interior; the appendix bursae is also smaller and more narrowly joined to the corpus bursae in *C.ochreata*. Compared to *C.albata*, overall shape and structure of the bursa copulatrix is very similar, but the spinules lining the inside of the bursae are smaller in *C.ochreata*.

#### Description.

***Head***: frons and vertex with mix of dirty-white and dull grey-brown scales; palpi grey brown, terminal (3^rd^) segment 0.5 × length of second segment; male antenna filiform and finely ciliate, segments approximately width 0.9 × that of length; dorsally with grey-brown scales, finely ciliate ventrally, with two mediolateral setae, these equal in length to antennal segment; female antenna similar to that of male but narrower and less ciliate. ***Thorax***: vestiture predominantly dirty white with scattered dull grey-brown scales; pro- and mesothoracic legs appearing striped, grey brown with a ring of dirty white scales at apices of segments, in addition to a mid-tibial pale ring; metathoracic leg entirely dirty white; metepisternal tymbal unscaled, with 9–11 fine grooves. ***Abdomen***: vestiture light grey brown dorsally and ventrally; females with apical tuft of incurved setae; males with a series of paired setal tufts ventrally on segments 7, 8 and 9, in addition to two paired lobe-like setal tufts situated within a pouch between segments 7 and 8. *Forewing*: ground color slightly ochre, dirty white; basal line indistinct greyish ochre; antemedial line double, often only with distal line defined; grey ochre; medial line grey ochre; postmedial line an irregular row of disconnected grey-black splotches; terminal line grey black, thin or incomplete, interrupted with white at vein termini; fringe dirty white, often interrupted with grey patch at apex and medial area; reniform spot usually very pronounced, grey black; orbicular spot small and grey black, often absent or tiny; ventrally with large dusky-grey patch medially, pattern inconspicuous, wing edges dull tan white, interrupted at costa by four costal grey-black markings; males with a brown setal (androconial?) tuft in medio-anal area; wing pattern of females similar, but usually with the dark markings reduced considerably, giving the impression of a paler moth. *Hindwing*: dirty white with faint, indistinctly delineated fuscous area in distal third; ventrally with slightly darker fuscous patch in anal angle and discal spot, and faint medial line. *Male genitalia* (Figure 15): Uncus attenuating towards base and to apex, with pronounced ventromedial bulge; apex acute, curved ventrad; long, thin setae in medial area, radiating outward; valval lobe consisting of an enlarged, flattened costa terminating in a small dorsally projecting apical spine, and a pronounced flange-like medial process, forming an evenly curved concave dorsal margin on the apical halve of the valve; valvule consisting of an indistinct, somewhat membranous lobe on the ventrodistal portion of the valve; sacullus with a flattened slightly spatulate dorsally projecting process; juxta poorly defined and not well sclerotized, shield shaped; phallus approximately 4 × longer than wide narrowing slightly subapically; vesica roughly kidney shaped with several lobe-like or globose diverticula; large cornutus situated medio-laterally on left side of vesica main chamber, approximately 2/3 length of phallus, vesica and diverticula finely spiculose. *Female genitalia* (Figure 18): papillae anales poorly sclerotized, relatively small, and slightly cupped; sparsely setose laterally and along caudal margin, dorsally with very fine, dense cilia-like setae caudal to opening of dorsal pheromone gland; anterior and posterior apophysis short, approximately equal in length to that of papillae; ductus bursae short and broad, about as long as wide and dorsoventrally flattened; ductus and corpus bursae joined by a smoothly sclerotized, broadly flat-conical chamber (cervix bursae); corpus bursae pear shaped but oriented laterally, i.e. narrowing into ductus seminalis to right; appendix bursae a globose bubble-like chamber situated proximally at base of corpus bursae; interior of appendix bursae and corpus bursae with dense field of spinules, in latter situated distally near juncture with cervix bursae; pleurite of A7 with shallow pockets, appearing somewhat rugose and more heavily sclerotized than surrounding integument.

#### Biology.

The immature stages and larval hosts are unknown, but larvae likely feed on algae or lichens growing on tree bark. There are multiple broods starting in March and continuing into September.

#### Distribution.

The Atlantic coastal plain from North Carolina southward into Florida and westward to eastern Texas (Figure 21).

#### Remarks.

The late Douglas Ferguson deemed *C.ochreata* to be closely related to the Mexican *C.patella* (Druce), and the latter name was therefore applied by him to this taxon (Lafontaine and Schmidt 2010). Examination of Mexican specimens of *C.patella* does indeed show that *patella* belongs to the *albata*-group, but the genitalic structure of *C.patella* is more divergent from the remaining members of this North American group.

**Figure 23.**
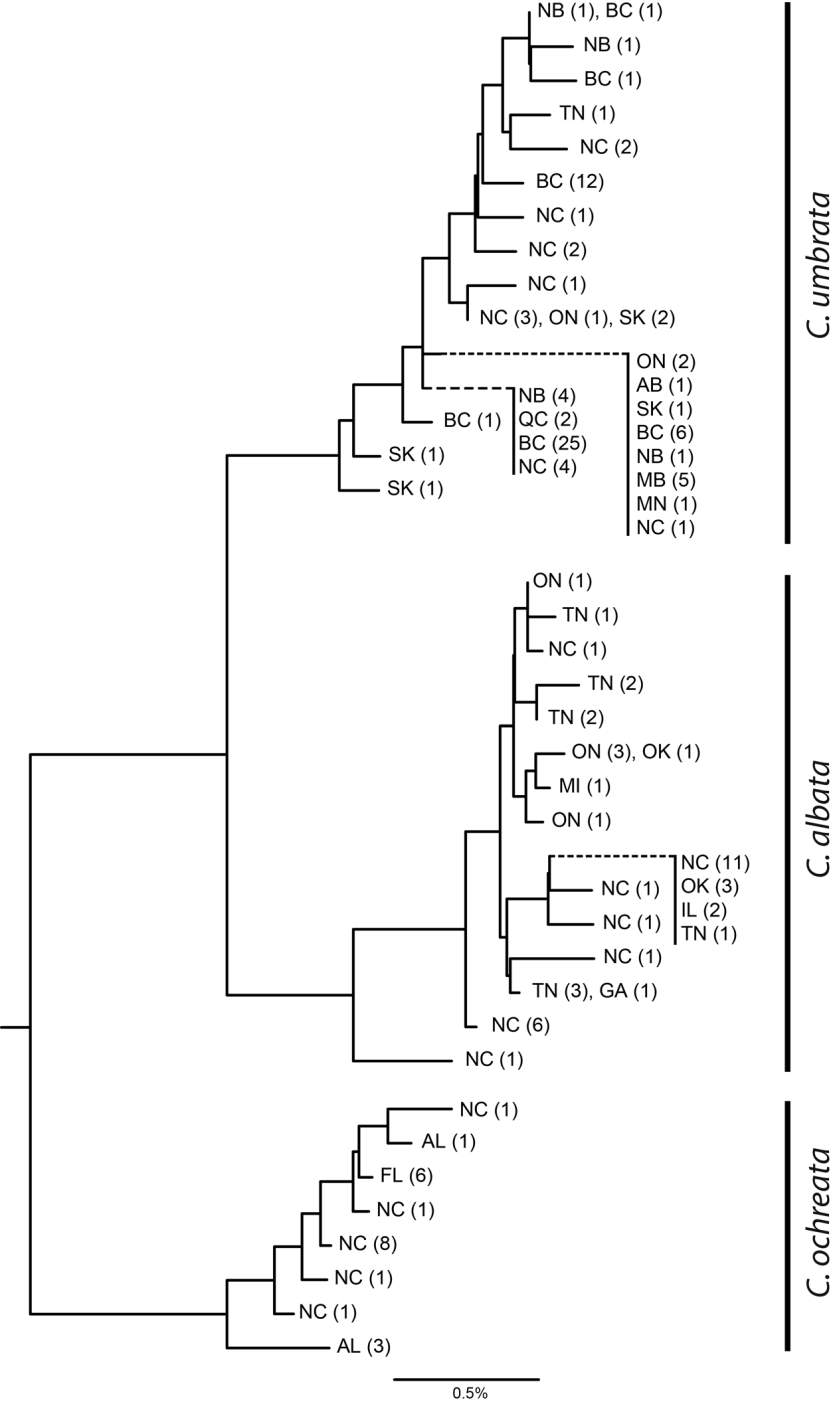
Neighbour-joining tree of representative mtDNA barcode haplotypes in North American *Clemensia* species. Sample size and locality are given in brackets, with number of specimens indicated after two-letter state/province abbreviation. *Lacinipoliasareta* variation is divided into five haplogroups, A–E. Voucher specimen data is given in Suppl. material 1.

## Conclusions

Although *Clemensiaalbata* has been treated as a single species, the concordant variation in phenotype, morphology, phenology distribution, and DNA barcode clearly supports a concept of three species. Further research is needed on *Clemensia* life histories, given the current taxonomic changes. Specifically, it is not known if the differences in voltinism and phenology correspond to differing winter diapause strategies, different food plant requirements or how the larvae differ morphologically. The distributional limits of the genus also requires refining, such as the northwestern range limits of *C.albata* and the full extent of the distribution of *C.umbrata* in the eastern US.

## Supplementary Material

XML Treatment for Clemensia umbrata

XML Treatment for Clemensia albata

XML Treatment for Clemensia ochreata

## Supplementary materials

Supplementary material 1 Table S1. DNA barcode voucher specimen data. Explanation note: Abbreviations for specimen depositories are as given in the Methods and Materials section. This is an open access article distributed under the terms of the Creative Commons Attribution License (CC BY 4.0), which permits unrestricted use, distribution, and reproduction in any medium, provided the original author and source are credited.B. Christian Schmidt, J. Bolling Sullivan
